# Role and regulation of Abelson tyrosine kinase in Crk-associated substrate/profilin-1 interaction and airway smooth muscle contraction

**DOI:** 10.1186/s12931-017-0709-4

**Published:** 2018-01-05

**Authors:** Yinna Wang, Alyssa C. Rezey, Ruping Wang, Dale D. Tang

**Affiliations:** Department of Molecular and Cellular Physiology, Albany Medical College, 47 New Scotland Avenue, MC-8, Albany, NY 12208 USA

**Keywords:** c-Abl kinase, Crk-associated substrate, Profilin-1, Smooth muscle, Excitation-contraction coupling, Actin cytoskeleton

## Abstract

**Background:**

Airway smooth muscle contraction is critical for maintenance of appropriate airway tone, and has been implicated in asthma pathogenesis. Smooth muscle contraction requires an “engine” (myosin activation) and a “transmission system” (actin cytoskeletal remodeling). However, the mechanisms that control actin remodeling in smooth muscle are not fully elucidated. The adapter protein Crk-associated substrate (CAS) regulates actin dynamics and the contraction in smooth muscle. In addition, profilin-1 (Pfn-1) and Abelson tyrosine kinase (c-Abl) are also involved in smooth muscle contraction. The interplays among CAS, Pfn-1 and c-Abl in smooth muscle have not been previously investigated.

**Methods:**

The association of CAS with Pfn-1 in mouse tracheal rings was evaluated by co-immunoprecipitation. Tracheal rings from c-Abl conditional knockout mice were used to assess the roles of c-Abl in the protein-protein interaction and smooth muscle contraction. Decoy peptides were utilized to evaluate the importance of CAS/Pfn-1 coupling in smooth muscle contraction.

**Results:**

Stimulation with acetylcholine (ACh) increased the interaction of CAS with Pfn-1 in smooth muscle, which was regulated by CAS tyrosine phosphorylation and c-Abl. The CAS/Pfn-1 coupling was also modified by the phosphorylation of cortactin (a protein implicated in Pfn-1 activation). In addition, ACh activation promoted the spatial redistribution of CAS and Pfn-1 in smooth muscle cells, which was reduced by c-Abl knockdown. Inhibition of CAS/Pfn-1 interaction by a decoy peptide attenuated the ACh-induced actin polymerization and contraction without affecting myosin light chain phosphorylation. Furthermore, treatment with the Src inhibitor PP2 and the actin polymerization inhibitor latrunculin A attenuated the ACh-induced c-Abl tyrosine phosphorylation (an indication of c-Abl activation).

**Conclusions:**

Our results suggest a novel activation loop in airway smooth muscle: c-Abl promotes the CAS/Pfn-1 coupling and actin polymerization, which conversely facilitates c-Abl activation. The positive feedback may render c-Abl in active state after contractile stimulation.

## Background

Upon contractile activation, myosin light chain undergoes phosphorylation at Ser-19, which initiates sliding of contractile filaments and smooth muscle contraction [1, 2]. In addition, actin filaments of smooth muscle connect with the extracellular matrix via the integrin-associated complex [3–6]. A pool of globular actin (G-actin) is added to existing filamentous actin (F-actin) in response to contractile stimulation, which promotes contraction by enhancing the transmission of force between the contractile units and the extracellular matrix [4, 6–10]. Thus, myosin can be viewed as an “engine” and the actin cytoskeleton as a “transmission system” for smooth muscle contraction [4, 11–14]. Although myosin activation has been extensively investigated [1, 2], the cellular processes that orchestrate actin polymerization are incompletely elucidated.

Proflin-1 (Pfn-1) is an actin-binding protein that promotes actin polymerization in vitro [15, 16], and endothelial cell migration and proliferation [17]. Pfn-1 promotes actin polymerization by increasing transport of actin monomers to existing actin filaments [8, 9, 16, 18]. Pfn-1 is involved in regulating smooth muscle functions including actin polymerization and contraction [19, 20]. Contractile stimulation promotes the coupling of cortactin (an actin-regulatory protein) with Pfn-1, which subsequently activates Pfn-1 and actin filament polymerization [4, 13]. However, other molecules may regulate the activation of Pfn-1 in smooth muscle.

CAS (Crk-associated substrate)/BCAR1 is a tyrosine-phosphorylated protein that has been implicated in actin reorganization, which is involved in a variety of cellular functions including smooth muscle contraction [9, 21–23]. In smooth muscle, CAS undergoes phosphorylation at Tyr-410 at substrate domain (SD) domain, which interacts with the SH2 domain of CrkII, and activates N-WASP and promotes actin filament polymerization [4, 8, 9, 21, 24].

c-Abl (Abelson tyrosine kinase) is a non-receptor tyrosine kinase that has been implicated in cell adhesion, migration, proliferation, cytokinesis, and smooth muscle contraction [21, 25–31]. c-Abl regulates smooth muscle contraction by modulating actin dynamics [21, 27, 31]. The interplays among CAS, Pfn-1 and c-Abl in smooth muscle have not been previously investigated.

In this report, we find that contractile activation enhances the coupling of CAS with Pfn-1, which is necessary for smooth muscle contraction. In addition, CAS/Pfn-1 coupling upon contractile activation is regulated by CAS phosphorylation. Furthermore, c-Abl promotes the interaction of CAS with Pfn-1 and actin dynamics in response to contractile stimulation.

## Methods

### Animals

All animal protocols were reviewed and approved by the Institutional Animal Care and Usage Committee (IACUC) of Albany Medical College. All experiments were strictly performed in accordance with approved protocols and regulations of IACUC. Animals were bred in the specific pathogen free housing of Animal Research Facility, Albany Medical College. The animal housing was kept at 21–22 °C with 45–55 relative humidity. The light/dark cycle of the housing was 7 am- 7 pm for fluorescent/LED lights and 7 pm – 7 am for red lights. The numbers of cage companions were 3–8 each based on animal ages and gender. Animals were healthy and able to breed normally. They were fed with Purina Lab Diet 5P76 and had continuous access to food and water.

c-Abl smooth muscle conditional knockout mice (c-Abl^smko^ mouse) and c-Abl^-lox^ mice were previously described [27]. c-Abl^-lox^ mice were a gift of Dr. Koleske of Yale University. SM22^cre^ mice were purchased from The Jackson Laboratory. c-Abl^-lox^ mice (genetic background, 129/Svj) were crossed with SM22^cre^ mice on C57BL/6 background. These mice express Cre recombinase under control of a smooth muscle-specific SM22 promoter. As a consequence, this loxP flanked exon 5 of the *abl* gene was excised in smooth muscle cells [27]. Genotyping and phenotyping for the mice were routinely performed by PCR and immunoblotting, respectively [27]. C57BL/6 mice were used for experiments associated with inhibitor treatment. Both male and female mice aging 10–20 weeks were randomized allocated to the experimental or control groups.

### Assessment of tracheal ring contraction

Mice were euthanized by intraperitoneal injection of euthanasia solution (VEDCO, 0.1 ml/25 g). A segment of tracheas (4–5 mm in length) was immediately removed and placed in physiological saline solution (PSS) containing 110 mM NaCl, 3.4 mM KCl, 2.4 mM CaCl2, 0.8 mM MgSO4, 25.8 mM NaHCO3, 1.2 mM KH2PO4, and 5.6 mM glucose. The solution was aerated with 95%O2–5%CO2 to maintain a pH of 7.4. Two stainless steel wires were passed through the lumen of tracheal rings. One of the wires was connected to the bottom of organ baths and the other was attached to a Grass force transducer that had been connected to a computer with A/D converter (Grass). Tracheal segments were then placed in PSS at 37 °C. At the beginning of each experiment, 0.5 g passive tension was applied to tracheal rings. After 60 min equilibrium they were stimulated with 80 mM KCl repeatedly until contractile responses and passive tension reached a steady state. Contractile force in response to acetylcholine was then measured.

### Cell culture

Human airway smooth muscle (HASM) cells were previously described by our laboratory [11–14, 28]. This study was approved by the Albany Medical College Committee on Research Involving Human Subjects. Cells were cultured at 37 °C in the presence of 5% CO_2_ in Ham’s F12 medium supplemented with 10% (*v*/v) fetal bovine serum (FBS) and antibiotics (100 units/ml penicillin, 100 μg/ml streptomycin). The medium was changed every 3–4 days until cells reached confluence, and confluent cells were passaged with trypsin/EDTA solution [28, 32, 33]. Smooth muscle cells within passage 5 were used for the studies.

Stable c-Abl knockdown (KD) HASM cells were generated as previously described [13, 26, 28, 29]. To construct lentivirus encoding c-Abl shRNA, oligonucleotides were synthesized by Invitrogen. The sense target sequence of c-Abl shRNA was 5’-AAGCCGCTCGTTGGAACTCCA-3′ (NCBI accession number NM_005231). Oligonucleotides with scramble sequence (5’-ATTGCTCATATTGGCTAT-3′) were used as control. Oligonucleotides encoding c-Abl or control shRNA were subcloned into pFUGW lentiviral vector followed by transformation into Stbl3 competent cells (Invitrogen). These constructs also carry the gene of green fluorescence protein (GFP) to monitor the infection efficiency of cells. The plasmid DNA was harvested and purified using the plasmid maxiprep kits (Invitrogen). To produce viruses, 293FTcells were transfected with pFUGW encoding c-Abl shRNA or control shRNA plus packaging vector pCMV and envelop vector pVSV-G. Viruses were collected 48 h after transfection. To infect cells, smooth muscle cells were incubated with viruses for 6 h. The cells were then incubated in the growth medium for 2–3 days. GFP fluorescence of infected cells was monitored by a digital fluorescent microscope. Infection rates were nearly 100% as evidenced by GFP fluorescence analysis [29]. Immunoblot analysis verified c-Abl KD in HASM cells [13, 26, 28].

### Immunoblot analysis

Pulverized tissues were lysed in SDS sample buffer composed of 1.5% dithiothreitol, 2% SDS, 80 mM Tris-HCl (pH 6.8), 10% glycerol and 0.01% bromophenol blue. The mixtures were boiled in the buffer for 5 min and separated by SDS-PAGE. Proteins were transferred to a nitrocellulose membrane. The membrane was blocked with bovine serum albumin or milk for 1 h and probed with use of primary antibody followed by horseradish peroxidase-conjugated secondary antibody (Fisher Scientific). Proteins were visualized by enhanced chemiluminescence (Fisher Scientific) using the GE Amersham Imager 600 system.

Antibodies used were CAS antibody (1:1000, BD Transduction Lab, #610272, L/N 3357634, 4,045,947 and 5,338,759), phospho-CAS (Y410) antibody (1:1000, Cell Signaling, #4011 s, L/N 2), Pfn-1 antibody (1:500, Sigma, #p7624, L/N 015m4753v; Santa Cruz, #sc-166,191, L/N 1–11,409), c-Abl antibody (1:500, Santa Cruz, #sc-23, L/N k0111 and #sc-56,887, L/N h1712), phospho-c-Abl (Y412) antibody (1:1000, Cell Signaling, #2865 s, L/N 3; Santa Cruz, #sc-101,626, L/N k0911), phospho-MLC (S19) antibody (1:1000, Santa Cruz, #sc-19,849-4), MLC antibody (1:3000, custom made) [34], α-actin antibody (1:3000, Sigma, #A2547, L/N 084m4795v), and cortactin antibody (1:1000, Santa Cruz, #SC-11408/LN F3010 and #SC-55578/LN Z0417). The levels of proteins were quantified by scanning densitometry of immunoblots (Fuji Multigauge Software or GE IQTL software). The luminescent signals from all immunoblots were within the linear range [12, 14, 28].

### Co-immunoprecipitation analysis

Co-immunoprecipitation analysis was used to evaluate protein-protein interactions as previously described [21, 33]. Briefly, tissue extracts were incubated overnight with corresponding antibodies and then incubated for 3 h with 150 μl of a 10% suspension of protein A-Sepharose beads. Immunocomplexes were washed four times in buffer containing 50 mM Tris-HCl (pH 7.6), 150 mM NaCl and 0.1% Triton X-100. The immunoprecipitates were separated by SDS-PAGE followed by transfer to nitrocellulose membranes. The membranes of immunoprecipitates were probed with use of corresponding antibodies.

### Immunofluorescent and fluorescent analysis

Cells or tissues were fixed for 15 min in 4% paraformaldehyde, and were then washed three times in phosphate-buffered saline (PBS) followed by permeabilization with 0.2% Triton X-100 dissolved in PBS for 5 min. These cells were immunofluorescently stained using specific antibodies followed by appropriate secondary antibodies conjugated with fluorophores (Invitrogen). The cellular localization of fluorescently labeled proteins was viewed under a high resolution digital fluorescent microscopy (Leica, 63× oil objective). The localization of labeled protein was also line scanned using Leica Image software. The time of image capturing, intensity gaining, and image contrast in both channels were optimally adjusted and kept constant for all experiments to standardize the fluorescence intensity measurements among experiments. For quantification analysis, cells with >50% of periphery displaying translocated proteins were considered translocated cells. The percentage of cells with translocation was calculated as follows: (number of cells with colocalization)/(number of total cells observed) × 100. Fluorophores used were rhodamine-phalloidin (Life Technologies, #R415, L/N 1738179) and Alexa fluor-488 DNase I (Life Technologies, #D12371, L/N 38965A).

### Preparation of a decoy peptide

A cell permeable decoy peptide (CAS) was designed to inhibit the interaction of CAS with Pfn-1. The sequence of CAS peptide was taken from the P-rich domain of CAS (sequence is GRKKRRQRRRPPQPPQPQPSLPQGVHAPVPP) (GeneBank number, U28151.1). The N-terminal end of the peptide was fused with TAT sequence (GRKKRRQRRRPPQ) for cell permeability. The peptide was synthesized and purified by Invitrogen. A peptide with scramble sequence (GRKKRRQRRRPPQQPLPQSPGPVPHPAVPQP**)** was also used as a control.

### DNA constructs and cell transfection

Constructs for WT CAS (wild type Crk-associated substrate) and CAS-SD (nonphosphorylatable Crk-associated substrate mutant) have been previously described [21, 35]. Constructs for WT cortactin (wild type cortactin) and NP cortactin (nonphosphorylatable cortactin mutant) have been previously described [26]. Cell transfection was performed by using the FuGENE HD transfection reagent kit (Promega) according to the manual of the manufacture.

### Analysis of F-actin/G-actin ratios

The content of F-actin and G-actin in smooth muscle was measured using a method previously described [21, 34, 36]. Briefly, smooth muscle cells were treated with F-actin stabilization buffer (50 mM PIPES, pH 6.9, 50 mM NaCl, 5 mM MgCl_2_, 5 mM EGTA, 5% glycerol, 0.1% Triton X-100, 0.1% Nonidet P40, 0.1% Tween 20, 0.1% beta-mercapto-ethanol, 1 mM ATP, 1 μg/ml pepstatin, 1 μg/ml leupeptin, 10 μg/ml benzamidine). The supernatants of protein extracts were collected after centrifugation at 151,000 g for 60 min at 37 °C. The pellets were resuspended in ice-cold H_2_O plus 1 μM cytochalasin D and then incubated on ice for 1 h to dissociate F-actin. The resuspended pellets were gently mixed every 15 min. The supernatant of the resuspended pellets was collected after centrifugation at 16,100 g for 2 min at 4 °C. Equal volume of the first supernatant (G-actin) or second supernatant (F-actin) was subjected to immunoblot analysis using α-actin antibody. The amount of F-actin and G-actin was determined by scanning densitometry.

### Statistical analysis

All statistical analysis was performed using Prism 6 software (GraphPad Software, San Diego, CA). Comparison among multiple groups was performed by two-way ANOVA followed by a post hoc test (Tukey’s multiple comparisons). Values of n refer to the number of experiments used to obtain each value. *P* < 0.05 was considered to be significant.

## Results

### Contractile activation increases the coupling of CAS with Pfn-1 in smooth muscle

**Fig. 1:**
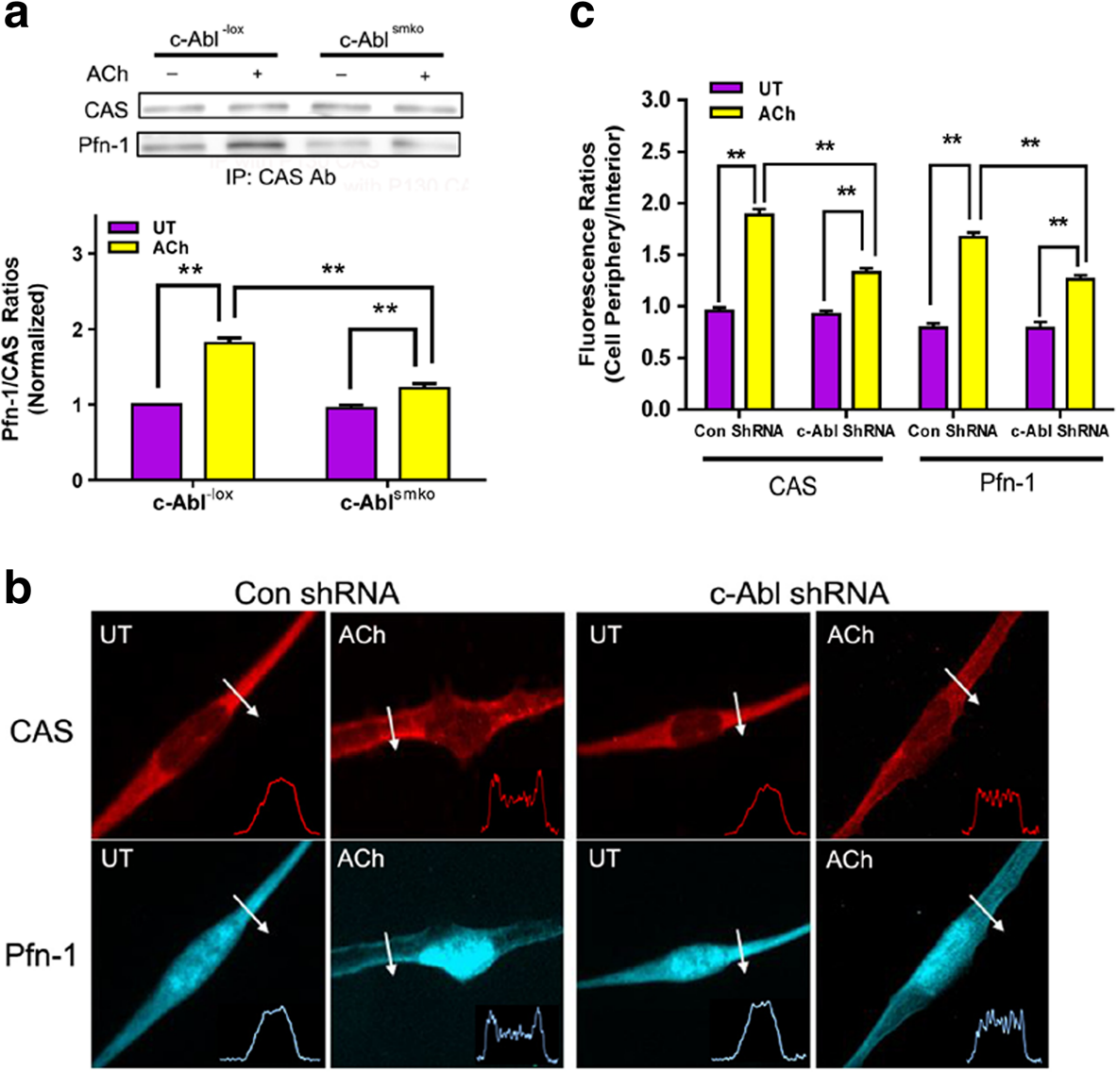
Increases in the association and translocation of CAS with Pfn-1 upon acetylcholine stimulation are regulated by c-Abl. **a** Mouse tracheal rings from c-Abl^-lox^ and c-Abl^smko^ mice were treated with acetylcholine (ACh) (100 μM, 5 min) or left untreated (UT) followed by CAS immunoprecipitation. CAS immunoprecipitates were separated by SDS-PAGE and blotted with antibodies against CAS and Pfn-1. Data are mean ± SE (*n* = 4). **b** Human airway smooth muscle (HASM) cells expressing control shRNA or c-Abl shRNA were stimulated with ACh (100 μM, 5 min) or left untreated. The cellular localization of CAS and Pfn-1 was evaluated by immunofluorescent microscopy. Arrows indicate a single line scan to analyze the fluorescent intensity for each cell. The inset plots represent fluorescent intensity of the line scan indicated by the arrows. **c** Fluorescent ratios of cell periphery over interior for CAS or Pfn-1 in cells were calculated. Data are mean ± SE (*n* = 26–27 cells from 4 independent experiments). UT, untreated. ** *P* < 0.01

CAS and Pfn-1 are involved in the regulation of actin reorganization and smooth muscle contraction [4, 6, 22, 37, 38]. The interaction of CAS with Pfn-1 has not been previously explored. We hypothesized that contractile stimulation may promote the association of CAS with Pfn-1, which may facilitate Pfn-1 mediated actin polymerization. To test this, wild type (WT) mouse tracheal rings were treated with acetylcholine (ACh), or left untreated. Tissues were immunoprecipitated with use of CAS antibody, and blotted with antibodies against CAS and Pfn-1. The amount of Pfn-1 in CAS immunoprecipitates was higher in stimulated tissues than in unstimulated rings. The protein ratios of Pfn-1/CAS were increased in stimulated tissues as compared to unstimulated rings (Fig. 1a). The results suggest that contractile activation promotes the interaction of CAS with Pfn-1 in smooth muscle.

### CAS and Pfn-1 translocate to the periphery of smooth muscle cells upon ACh stimulation

Because actin polymerization occurs at cortical regions in smooth muscle upon contractile activation [4, 6, 10], we also used immunofluorescent microscopy to assess the spatial localization of CAS and Pfn-1. CAS was localized in the cytoplasm of unstimulated human airway smooth muscle (HASM) cells. CAS redistributed to the cortical region of cells in response to ACh activation. Pfn-1 was localized in the cytoplasm and the nucleus of unstimulated cells. Upon ACh stimulation, Pfn-1 was able to translocate to the cell periphery (Fig. 1b and c).

### Interaction of CAS with Pfn-1 is inhibited by c-Abl knockout

Because c-Abl is a non-receptor tyrosine kinase that regulates actin dynamics and the contraction in smooth muscle [3, 4, 21, 25, 27, 31], we evaluated whether c-Abl affects the coupling of CAS with Pfn-1 by assessing the effects of c-Abl smooth muscle conditional knockout (KO) [27] on CAS/Pfn-1 coupling using co-immunoprecipitation. The ACh-induced increase in CAS/Pfn-1 coupling was reduced in tissues from c-Abl^smko^ mice as compared to c-Abl^-lox^ mice (Fig. 1a). Moreover, c-Abl knockdown (KD) also reduced the ACh-induced CAS and Pfn-1 translocation to the cortical region (Fig. 1b and c).

### Effects of c-Abl KO on actin polymerization, contraction, myosin activation and CAS phosphorylation

**Fig. 2:**
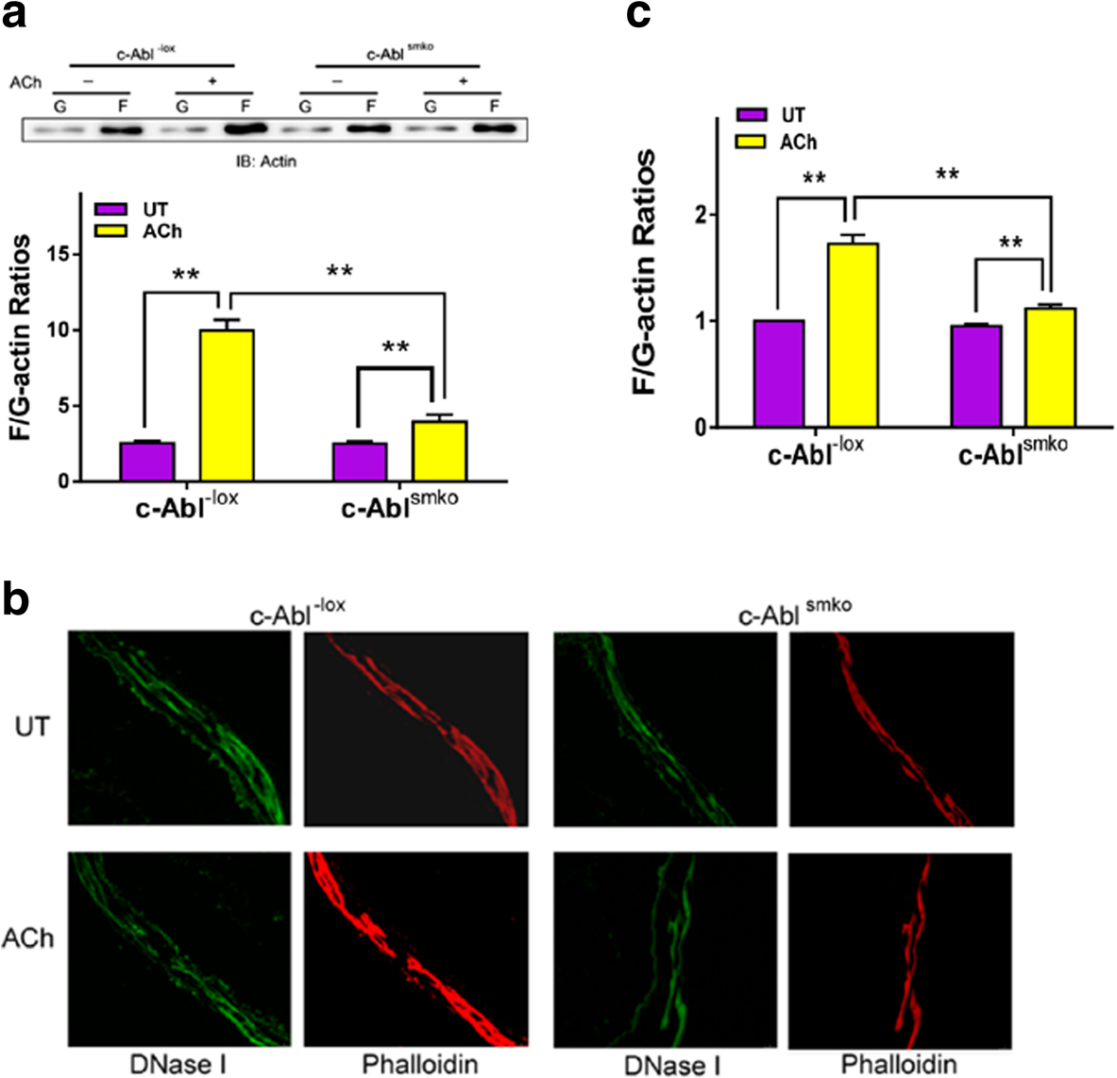
c-Abl knockout inhibits actin filament polymerization in response to ACh stimulation. **a** Mouse tracheal rings from c-Abl^-lox^ and c-Abl^smko^ mice were treated with acetylcholine (ACh) (100 μM, 5 min) or left untreated (UT). F/G-actin ratios were evaluated using the fractionation assay. Data are mean ± SE (*n* = 5). **b** Representative images illustrating the effects of c-Abl knockout on F/G-actin ratios. Sections of trachealis from c-Abl^-lox^ and c-Abl^smko^ mice were stained with DNase I (for G-actin) or phalloidin (for F-actin). **c** ACh-induced increases in F/G-actin ratios evaluated by fluorescent microscopy are reduced in c-Abl^smko^ mice (*n* = 4). *P < 0.05, **P < 0.01

**Fig. 3:**
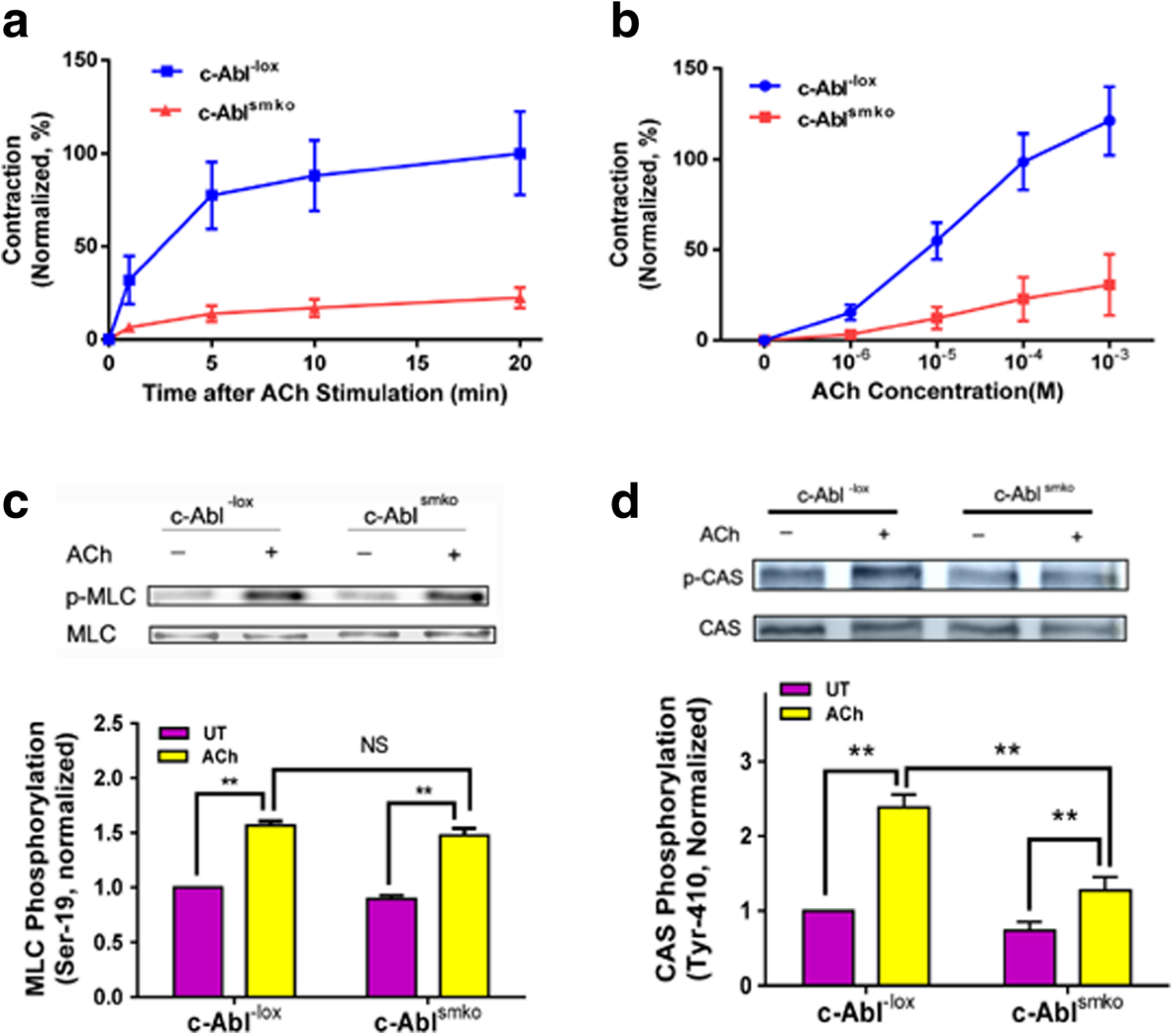
Smooth muscle contraction and CAS phosphorylation, but not myosin light chain phosphorylation, are regulated by c-Abl. **a** & **b** Contractile force was reduced in tissues from c-Abl^smko^ than in c-Abl^-lox^ mice, which is time- and dose-dependent (*n* = 5–7). Contractile force is normalized to the force induced by 10^−4^ M ACh. **c** Immunoblot analysis was used to assess myosin light chain phosphorylation at Ser-19 of extracts from tracheal rings of c-Abl^-lox^ and c-Abl^smko^ mice. Data are mean ± SE (*n* = 4). **d** ACh-induced CAS phosphorylation at Tyr-410 was reduced in tracheal rings from c-Abl^smko^ mice as compared to c-Abl^-lox^ mice. Data are mean ± SE (*n* = 6). **P < 0.01. NS, not significant

Because c-Abl KO inhibits the association of CAS with Pfn-1, we determined whether c-Abl KO affects actin polymerization, myosin light chain phosphorylation, and contraction. c-Abl KO diminished ACh-induced F/G-actin ratios evaluated by the fractionation assay (Fig. 2a) and fluorescent microscopy (Fig. 2b and c). Furthermore, c-Abl was required for airway smooth muscle contraction. c-Abl KO attenuated tracheal contraction in a time- and dose-dependent manner (Fig. 3a and b). However, myosin light chain phosphorylation at Ser-19 was not affected by c-Abl KO (Fig. 3c). Since CAS undergoes phosphorylation in smooth muscle in response to contractile activation, we determined the role of c-Abl in CAS phosphorylation. The ACh-induced CAS phosphorylation at Tyr-410 was reduced by c-Abl KO (Fig. 3d).

### Inhibition of CAS/Pfn-1 coupling attenuates actin polymerization and contraction without affecting myosin phosphorylation

**Fig. 4:**
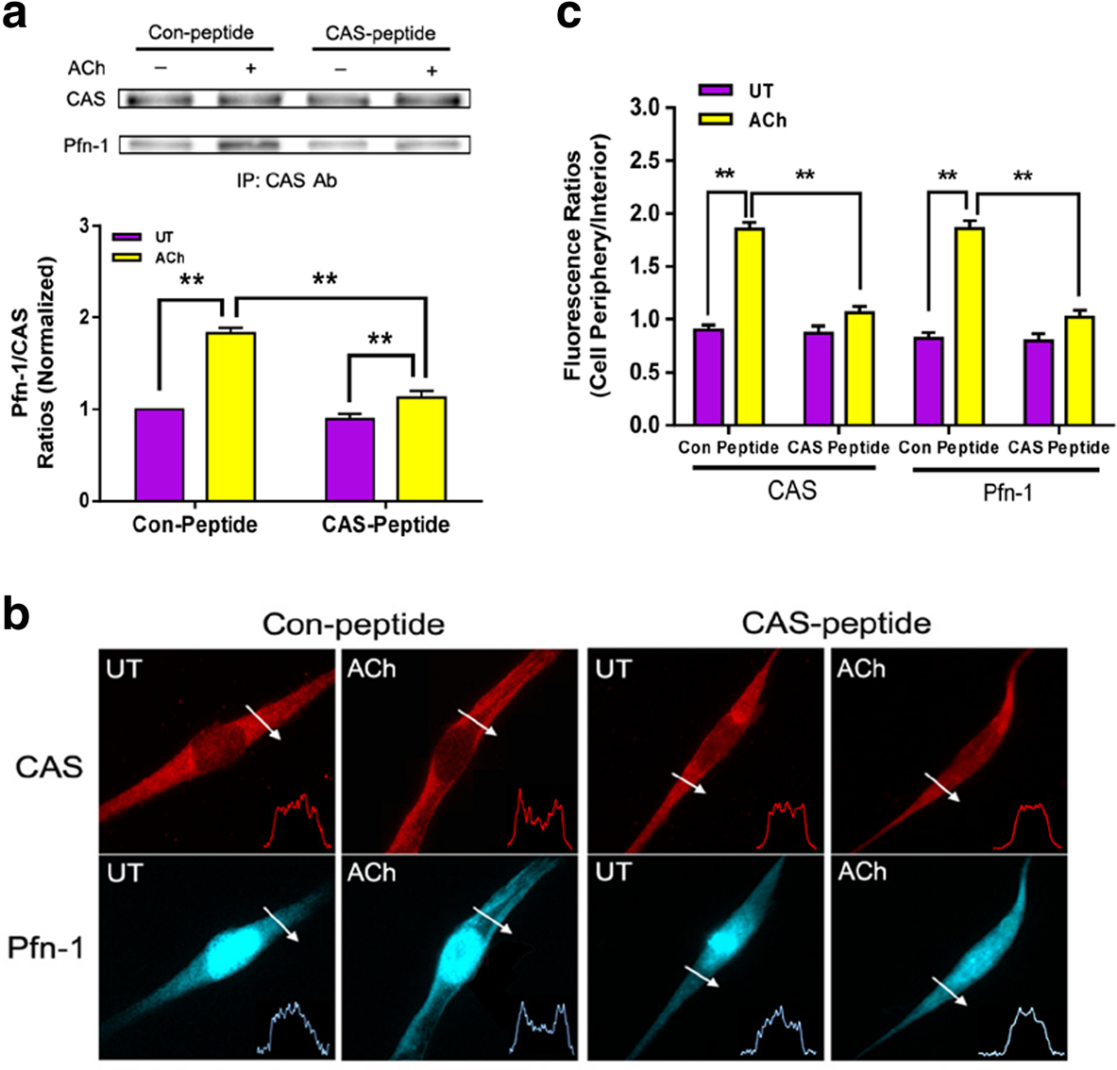
Treatment with CAS peptide attenuates CAS/Pfn-1 coupling and spatial redistribution of CAS and Pfn-1 upon contractile stimulation. **a** Mouse tracheal rings were pretreated with control (Con) or CAS peptide (2.5 μg/ml) for 30 min. They were then stimulated with ACh (100 μM, 5 min), or left untreated (UT). The protein-protein interaction was evaluated by co-immunoprecipitation. Data are mean ± SE (*n* = 4). **b** HASM cells pretreated with control or CAS peptide were stimulated with ACh (100 μM, 5 min), or were untreated. The spatial localization of CAS and Pfn-1 in the cells was assessed by immunostaining. Arrows indicate a single line scan to analyze the fluorescent intensity for each cell. The inset plots represent fluorescent intensity of the line scan indicated by the arrows. **c** Fluorescent ratios of cell periphery over interior for CAS or Pfn-1 in cells were calculated. Data are mean ± SE (*n* = 26 cells from 4 independent experiments). **P < 0.01. UT, untreated

**Fig. 5:**
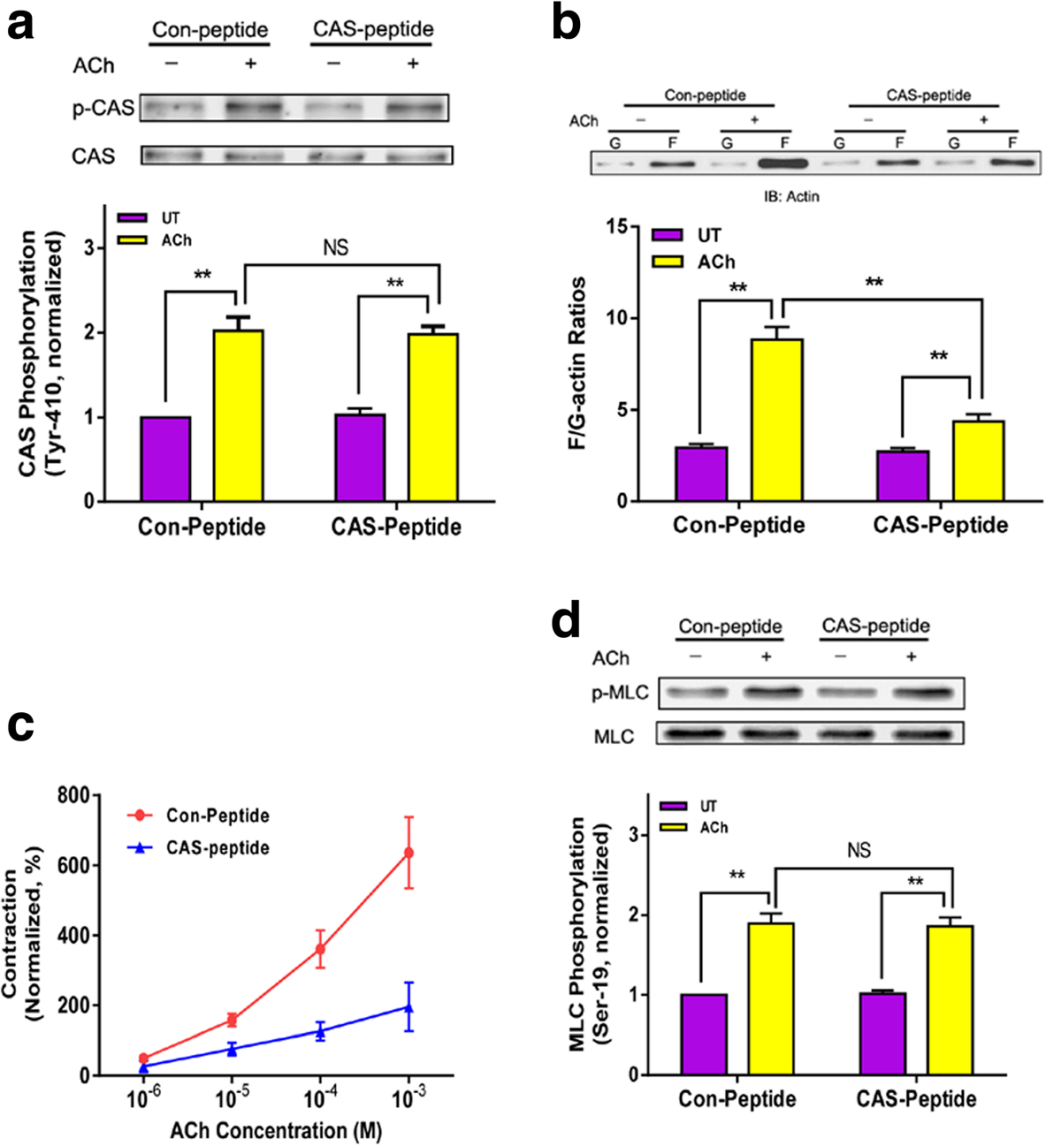
Effects of CAS-peptide on CAS phosphorylation, F/G-actin ratios, contraction and myosin phosphorylation. **a** Mouse tracheal rings pretreated with control or CAS peptide were stimulated with ACh (100 μM, 5 min), or left unstimulated. CAS phosphorylation at Tyr-410 was determined by immunoblot analysis. Data are mean ± SE (*n* = 4). **b** Mouse tracheal tissues that had been pretreated with control or CAS peptide were stimulated with ACh or left untreated. F/G-actin ratios in tissues were evaluated using the fractionation assay. Values represent mean ± SE (*n* = 5). **c** Contractile response of mouse tracheal rings to ACh (10^−5^ M) was determined, after which they were treated with peptides (2.5 μg/ml) for 30 min. They were then stimulated with different concentration of ACh. Contractile force is normalized to contraction induced by 10^−5^ M ACh before treatment with the peptides (*n* = 7–8). **d** Myosin light chain phosphorylation in mouse tracheal segments pretreated with peptides was assessed by immunoblot analysis. Values represent mean ± SE (*n* = 4). **P < 0.01; NS, not significant

Co-immunoprecipitation analysis was utilized to evaluate the effects of CAS-peptide on CAS/Pfn-1 interaction. The amount of Pfn-1 co-immunoprecipitated with CAS during ACh stimulation was lower in tissues treated with CAS-peptide than in tissues treated with scramble (control) peptide (Fig. 4a). Moreover, translocation of Pfn-1 and CAS to the cell edge in response to ACh activation was reduced in cells treated with CAS-peptide compared to cells treated with control peptide (Fig. 4b and c). However, CAS phosphorylation during ACh stimulation was not affected by CAS peptide (Fig. 5a). The results suggest that CAS peptide selectively hinders the coupling of CAS with Pfn-1.

To determine the functional role of CAS/Pfn-1 coupling, WT mouse tracheal rings were treated with peptides for 30 min. They were then stimulated with ACh or left unstimulated. F/G-actin ratios in these tissues were determined using the fractionation assay. F/G-actin ratios upon contractile activation were reduced in tissues treated with CAS-peptide compared to cells treated with control peptide (Fig. 5b). Furthermore, we determined the effects of CAS-peptide on smooth muscle contraction. Contractile force was attenuated in mouse tracheal rings treated with CAS-peptide (Fig. 5c). However, myosin light chain phosphorylation was not affected by CAS-peptide (Fig. 5d).

### CAS phosphorylation regulates its binding to Pfn-1

**Fig. 6:**
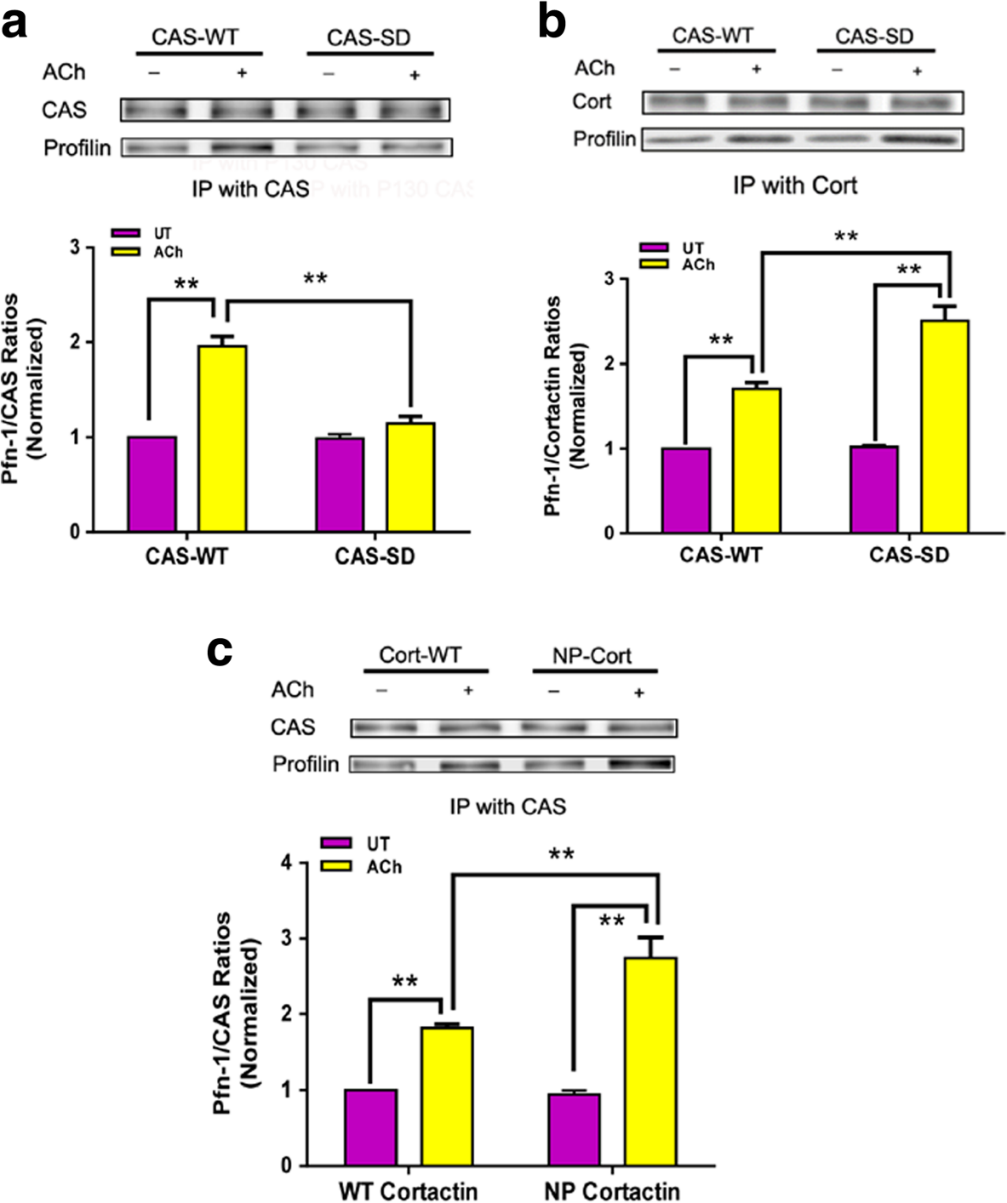
CAS/Pfn-1 coupling and cortactin/Pfn-1 interaction are regulated by phosphorylation. **a** HASM cells expressing wild type (WT) CAS and CAS-SD mutant were stimulated with ACh (100 μM, 5 min), or left unstimulated. The protein-protein interaction was evaluated by co-immunoprecipitation. Data are mean ± SE (*n* = 4). **b** ACh-induced interaction of cortactin with Pfn-1 is enhanced in cells expressing non-phosphorylated CAS mutant. Cortactin/Pfn-1 interaction was evaluated for HASM cells expressing CAS-WT and CAS-SD. Data are mean ± SE (*n* = 4). **c** Association of CAS with Pfn-1 upon ACh stimulation is increased in cells expressing non-phosphorylated cortactin mutant. CAS/Pfn-1 coupling was assessed for HASM cells expressing cortactin-NP. Values are mean ± SE (*n* = 4). NP, non-phosphorylated; cort, cortactin. **P < 0.01

Because contractile activation increases CAS phosphorylation at Tyr-410 and CAS/Pfn-1 interaction, we further questioned whether CAS phosphorylation affects its interaction with Pfn-1. HASM cells were transfected with constructs for WT CAS and CAS-SD (a CAS mutant without Tyr-410). Co-immunoprecipitation analysis was utilized to evaluate the association of CAS with Pfn-1. ACh stimulation increased Pfn-1/CAS protein ratios in cells expressing WT CAS. In contrast, ACh-induced increases in Pfn-1/CAS ratios were reduced in cells expressing CAS-SD (Fig. 6a). The results indicate that CAS phosphorylation controls its coupling with Pfn-1.

### Phosphorylated CAS affects cortactin/Pfn-1 coupling

As described earlier, phosphorylated cortactin also increases its coupling with Pfn-1 upon contractile stimulation [3, 4, 13]. This raises the possibility that phosphorylated CAS may affect the protein-protein interaction. To test this, we assessed the effects of non-phosphorylated CAS on the association of cortactin with Pfn-1. In cells expressing WT CAS, ACh stimulation increased the coupling of cortactin with Pfn-1. Moreover, the ACh-induced cortactin/Pfn-1 coupling in cells expressing CAS-SD was higher than cells expressing WT-CAS (Fig. 6b).

### Phosphorylated cortactin modulates CAS/Pfn-1 interaction

We also evaluated the influence of non-phosphorylated cortactin on CAS/Pfn-1 interaction. The association of CAS with Pfn-1 upon ACh stimulation was enhanced in cells expressing non-phosphorylated (NP) cortactin mutant than in cells expressing WT cortactin (Fig. 6c).

### C-Abl phosphorylation is regulated by Src during contractile activation

**Fig. 7:**
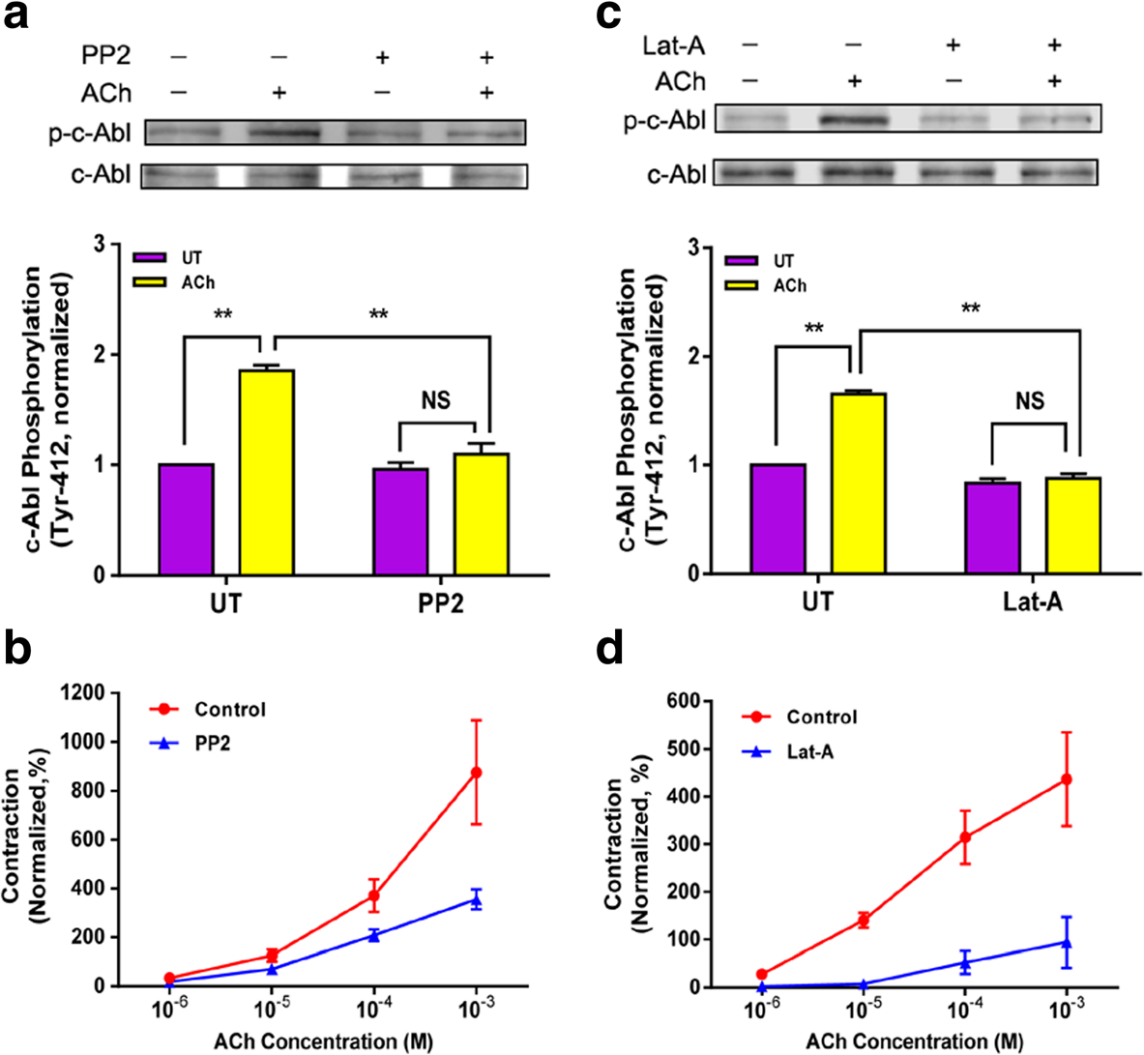
c-Abl phosphorylation is regulated by Src and actin polymerization. **a** Mouse tracheal rings pretreated with the Src inhibitor PP2 (10 μM, 30 min) were stimulated with ACh or unstimulated. c-Abl phosphorylation was evaluated by immunoblotting. Data are mean ± SE (*n* = 4). **b** Treatment with PP2 attenuates contractile response of tracheal segments to ACh (*n* = 4–6). Contractile force is normalized to the force induced by 10^−5^ M ACh before addition of PP2. **c** Immunoblotting was used to evaluate c-Abl phosphorylation of unstimulated and stimulated mouse tracheal rings pretreated with the actin polymerization inhibitor latrunculin A (1 μM, 30 min). (*n* = 4). **d** Contraction of mouse tracheal rings were reduced by treatment with latrunculin A (Lat-A). Contractile force is normalized to the force induced by 10^−5^ M ACh before addition of Lat-A. **P < 0.01; NS, not significant

c-Abl undergoes phosphorylation at Tyr-412 in smooth muscle in response to contractile activation, an indication of c-Abl activation [21, 39]. Because Src tyrosine kinase has been implicated in regulating c-Abl in fibroblasts [4, 40], we assessed the effects of the Src inhibitor PP2 on c-Abl phosphorylation. Treatment with PP2 attenuated the ACh-induced c-Abl phosphorylation (Fig. 7a). Furthermore, contractile response to ACh was reduced in tissues treated with PP2 (Fig. 7b).

### Inhibition of actin polymerization diminishes c-Abl phosphorylation during contractile activation

Because actin dynamics conversely regulates equatorial localization of c-Abl during cell division [4, 26], we examined whether actin polymerization affects c-Abl phosphorylation by determining the effects of the actin polymerization inhibitor latrunculin A on c-Abl phosphorylation. Exposure to latrunculin A inhibited the ACh-induced c-Abl phosphorylation in smooth muscle (Fig. 7c). Moreover, treatment with latrunculin A diminished contractile response to ACh (Fig. 7d). The results suggest that actin filament polymerization may conversely promote c-Abl activation.

## Discussion

In response to external stimulation, CAS undergoes phosphorylation at Tyr-410, which interacts with the SH2 domain of CrkII, and activates N-WASP and promotes actin filament polymerization [4, 8, 9, 21, 24]. In this report, we discover a new CAS-mediated downstream cascade. Contractile activation enhanced the association of CAS with Pfn-1, and their translocation to the cortical region. In addition, disruption of CAS/Pfn-1 coupling attenuated actin polymerization and contraction without affecting myosin activation. These studies suggest that the association of CAS with Pfn-1 in the near membrane region is important for actin dynamics and smooth muscle contraction. The binding of CAS to Pfn-1 may be mediated by the interaction of proline-rich region of CAS with the binding cleft on Pfn-1 [13, 41]. Moreover, the coupling of CAS with Pfn-1 may activate Pfn-1, which may catalyze the exchange of actin-bound ADP for ATP and release actin monomer from thymosin-β4; both processes facilitate unidirectional addition of G-actin to F-actin [3, 4, 8, 9, 18].

Here, we provide the first evidence to suggest that CAS/Pfn-1 interaction serves as a molecular switch to regulate actin dynamics and the contraction in smooth muscle. Moreover, non-phosphorylatable CAS mutant inhibited the ACh-induced association of CAS with Pfn-1. The results suggest that the CAS/Pfn-1 interaction is regulated by CAS tyrosine phosphorylation. Our previous studies suggest that cortactin phosphorylation is able to change its conformation and thus affect the interaction of cortactin with Pfn-1 [13]. It is likely that CAS phosphorylation may alter its structural conformation and enhance its association with Pfn-1.

Our previous studies have shown that cortactin promotes actin polymerization in smooth muscle in response to agonist stimulation [13]. In addition, contractile activation also increases the coupling of cortactin with Pfn-1 upon contractile stimulation [3, 4, 13]. In this report, expression of the non-phosphorylated CAS mutant increased cortactin/Pfn-1 coupling whereas the non-phosphorylated cortactin mutant enhanced CAS/Pfn-1 interaction. One possibility is that phosphorylated CAS and phosphorylated cortactin are able to compete for Pfn-1 occupancy. This competition for Pfn-1 residence may provide a redundant mechanism for Pfn-1 activation.

In this study, c-Abl conditional knockout attenuated CAS phosphorylation, CAS/Pfn-1 interaction, actin dynamics, and smooth muscle contraction during contractile activation. The results suggest that c-Abl plays an important role in regulating the CAS/Pfn-1 cascade and actin dynamics in smooth muscle.

Actin polymerization may facilitate smooth muscle contraction by several mechanisms. First, the actin filaments of smooth muscle cells connect to the cytoplasmic domain of β integrins via linker proteins such as vinculin, talin and α-actinin whereas the extracellular portion of β integrins engages with the extracellular matrix [6, 8, 9, 42, 43]. Thus, the β integrin-associated complex is able to transmit mechanical force between the contractile unit and the extracellular matrix [6, 8, 9, 42–44]. Nascent actin polymerization transpires at the near membrane region of smooth muscle [6, 8, 9, 34, 36, 42, 43], which may strengthen the engagement of actin filaments to β integrins and enhance the transmission of contractile force [5, 6, 8–10, 43, 44]. Second, actin filament assembly may participate in the “latch bridge” formation of contractile filaments, supporting force maintenance under the condition of lower crossbridge phosphorylation [7, 8, 45, 46]. Third, our recent studies suggest that actin polymerization promotes the recruitment of β-catenin to N-cadherin, which may facilitate the cell-to-cell force transmission and contraction [3, 4, 12].

c-Abl phosphorylation at Tyr-412 increases its kinase activity [14, 39]. Tyr-412 is located at the activation loop of c-Abl kinase domain. When inactive, the activation loop of the c-Abl kinase domain folds into the active site, thereby preventing binding of both substrates and ATP. Phosphorylation at Tyr-412 induces conformation changes; the activation loop no longer blocks the active site, which leads the increase in kinase activity [14, 39, 47].

Previous studies have shown that c-Abl may undergo autophosphorylation and activation upon external stimulation [39, 47]. c-Abl autophosphorylation may be promoted by its interaction with other proteins such as the adapter protein Abi1 and the Ras effector protein RIN1 [3, 4, 14, 40]. Our present results suggest that c-Abl phosphorylation at Tyr-412 is mediated by c-Src, which is supported by prior studies on fibroblasts [4, 40]. More importantly, treatment with the actin polymerization inhibitor attenuated c-Abl phosphorylation. The results indicate that actin filament polymerization may conversely uphold c-Abl activation. The mechanisms by which actin polymerization promotes c-Abl activation are currently unknown. Since Abi1 and RIN1 promote c-Abl activation [3, 4, 14, 40], it is likely that actin polymerization may facilitate Abi1 or RIN1-mediated c-Abl activation. Future studies are required to test the possibility.

## Conclusions

**Fig. 8:**
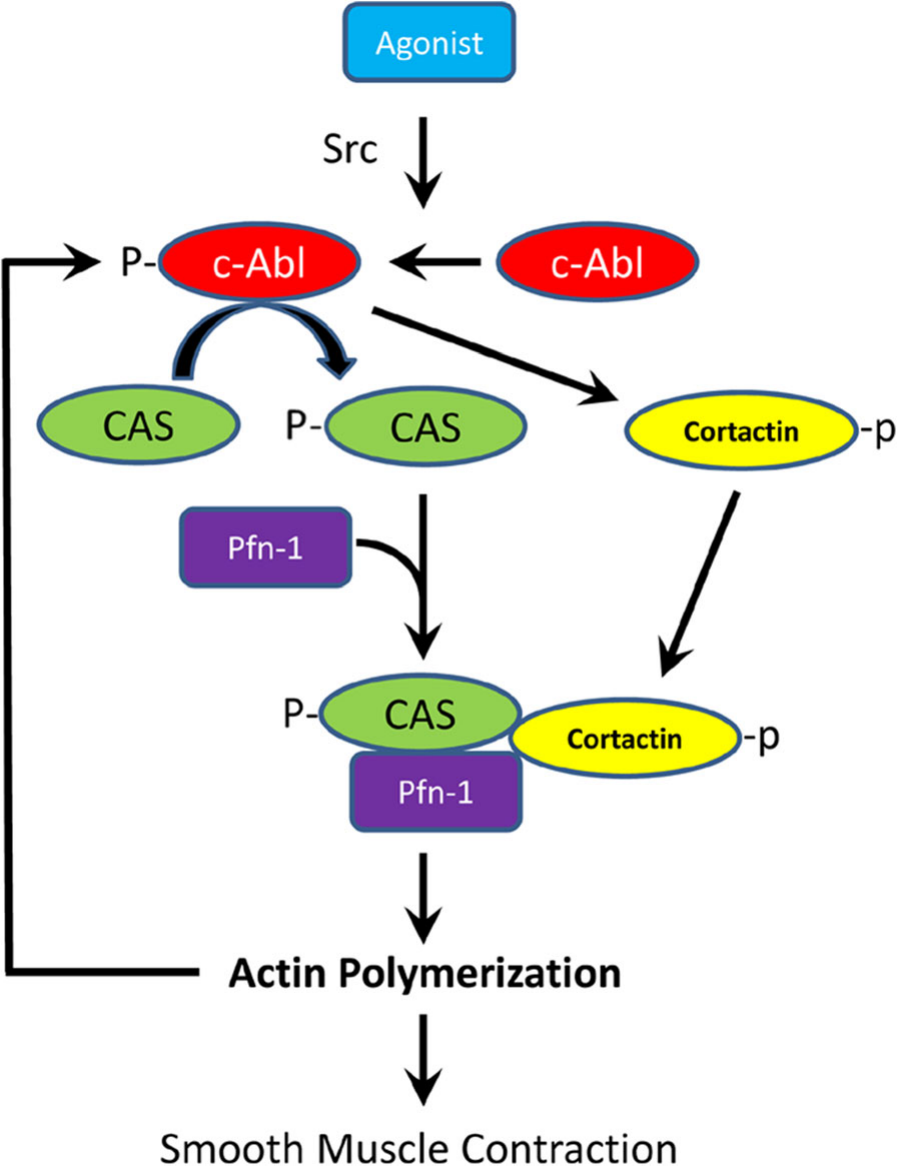
Novel mechanisms for regulation of actin dynamics. Upon agonist activation, c-Abl mediates CAS phosphorylation, which promotes the association of CAS with Pfn-1 to activate Pfn-1. In addition, c-Abl also catalyzes cortactin phosphorylation and increases the coupling of cortactin with Pfn-1. Activated Pfn-1 facilitates actin polymerization. Src mediates c-Abl activation in response to contractile stimulation. Furthermore, actin polymerization provides a positive feedback for c-Abl activation

Our previous studies demonstrate that c-Abl promotes cortactin phosphorylation, cortactin/Pfn-1 coupling and actin dynamics in smooth muscle in response to agonist stimulation [4, 13]. In the present study, contractile stimulation promotes the interaction of CAS with Pfn-1 in smooth muscle, which contributes to the regulation of smooth muscle contraction. CAS/Pfn-1 coupling is regulated by c-Abl tyrosine kinase. Furthermore, c-Abl activation is mediated by Src and increased by F-actin. Thus, we propose a novel activation loop: c-Abl promotes actin polymerization through the CAS/Pfn-1 pathway. Actin polymerization conversely facilitates c-Abl activation (Fig. 8). The positive feedback may render c-Abl in active state after contractile stimulation [14].

## Abbreviations

ACh: Acetylcholine
c-Abl: Abelson tyrosine kinase
CAS: Crk-associated substrate
GAPDH: Glyceraldehyde 3-phosphate dehydrogenase
HASM: Human airway smooth muscle
KD: Knockdown
KO: Knockout
MLC: Myosin light chain
N-WASP: Neuronal Wiskott–Aldrich Syndrome Protein
Pfn-1: Profilin-1
SH2: Src homology 2
shRNA: short hairpin RNA
WT: Wild type
